# Supplementary material for: Comparison of regional with general anaesthesia on postoperative delirium (RAGA-delirium) in the older patients undergoing hip fracture surgery: study protocol for a multicentre randomised controlled trial
Source: BMJ Open. 2017 Oct 22;7(10):e016937. doi: 10.1136/bmjopen-2017-016937 (PMC5665328; doi:10.1136/bmjopen-2017-016937)
Supplement: Author's manuscript [file bmjopen-2017-016937.draft_revisions.pdf]

BMJ Open

BMJ Open is committed to open peer review. As part of this commitment we make the peer review history of every article we publish publicly available.

When an article is published we post the peer reviewers' comments and the authors' responses online. We also post the versions of the paper that were used during peer review. These are the versions that the peer review comments apply to.

The versions of the paper that follow are the versions that were submitted during the peer review process. They are not the versions of record or the final published versions. They should not be cited or distributed as the published version of this manuscript.

BMJ Open is an open access journal and the full, final, typeset and author-corrected version of record of the manuscript is available on our site with no access controls, subscription charges or pay-per-view fees (http://bmjopen.bmj.com).

If you have any questions on BMJ Open's open peer review process please email editorial.bmjopen@bmj.com

BMJ Open

Comparison of regional with general anaesthesia on post-operative delirium (RAGA-delirium) in the older patients undergoing hip fracture surgery: study protocol for a multicentre randomized controlled trial

Journal:	*BMJ Open*
Manuscript ID	bmjopen-2017-016937
Article Type:	Protocol
Date Submitted by the Author:	20-Apr-2017
Complete List of Authors:	Li, Ting; The Second Affiliated Hospital and Yuying Children hospital of Wenzhou Medical University<br>Yeung, Joyce; University of Birmingham, College of Medical and Dental Sciences; Heart of England NHS Foundation Trust, Academic Department of Anaesthesia, Critical Care, Pain and Resuscitation<br>Li, Jun; The Second Affiliated Hospital and Yuying Children hospital of Wenzhou Medical University<br>Zhang, Yan; Fudan University; Shanghai Institute of Planned Parenthood Research (SIPPR)<br>Melody, Teresa; Heart of England NHS Foundation Trust, Academic Department of Anaesthesia, Critical Care, Pain and Resuscitation<br>Gao, Ye; The Second Affiliated Hospital and Yuying Children hospital of Wenzhou Medical University<br>Wang, Yi; The Second Affiliated Hospital and Yuying Children hospital of Wenzhou Medical University<br>Lian, Qian-Quan; The Second Affiliated Hospital and Yuying Children hospital of Wenzhou Medical University<br>Gao, Fang; The Second Affiliated Hospital and Yuying Children hospital of Wenzhou Medical University; University of Birmingham, Institute of Inflammation and Ageing, College of Medical and Dental Sciences
**Primary Subject Heading**:	Anaesthesia
Secondary Subject Heading:	Geriatric medicine
Keywords:	Hip fracture, Postoperative delirium, general anaestheia, regional anaesthesia

SCHOLARONE™ Manuscripts

TITLE: Comparison of regional with general anaesthesia on post-operative delirium (RAGA-delirium) in the older patients undergoing hip fracture surgery: study protocol for a multicentre randomized controlled trial

AUTHORS: Ting Li, Joyce Yeung, Jun Li, Yan Zhang, Teresa Melody, Ye Gao, Yi Wang, Qianquan Lian, Fang Gao*, on behalf of the RAGA-Delirium Investigators

Ting Li:
1 The Second Affiliated Hospital and Yuying Children hospital of Wenzhou Medical University, Wenzhou, Zhejiang, CN

Joyce Yeung:
1 University of Birmingham, College of Medical and Dental Sciences, Birmingham, UK
2. Heart of England NHS Foundation Trust, Academic Department of Anaesthesia, Critical Care, Pain and Resuscitation, Birmingham Heartlands Hospital, Birmingham, UK

Jun Li:
1 The Second Affiliated Hospital and Yuying Children hospital of Wenzhou Medical University, Wenzhou, Zhejiang, CN

Yan Zhang:
1. Fudan University, Shanghai, CN
2. Shanghai Institute of Planned Parenthood Research (SIPPR),Shanghai, CN

Teresa Melody:
1. Heart of England NHS Foundation Trust, Academic Department of Anaesthesia, Critical Care, Pain and Resuscitation, Birmingham, UK

Ye Gao:
1 The Second Affiliated Hospital and Yuying Children hospital of Wenzhou Medical University, Wenzhou, Zhejiang, CN

Yi Wang:
1 The Second Affiliated Hospital and Yuying Children hospital of Wenzhou Medical University, Wenzhou, Zhejiang, CN

Qianquan Lian:
1 The Second Affiliated Hospital and Yuying Children hospital of Wenzhou Medical University, Wenzhou, Zhejiang, CN

Prof. Fang Gao (*corresponding author):
1. The Second Affiliated Hospital and Yuying Children hospital of Wenzhou Medical University, Wenzhou, Zhejiang, CN

For peer review only

For peer review only - http://bmjopen.bmj.com/site/about/guidelines.xhtml

2. University of Birmingham, Institute of Inflammation and Ageing, College of Medical and Dental Sciences, Birmingham, UK
Postal Address: Queen Elizabeth Hospital, University of Birmingham, Birmingham, B15 2WB, UK
Tel: +44 (0) 121 424 2966
Fax: +44 (0) 121 424 2971
Email: F.GaoSmith@bham.ac.uk

ABSTRACT

Introduction:
Postoperative delirium (POD) is a common serious postoperative complication especially in older people, and is associated with increased mortality, morbidity and healthcare cost. There is no clear consensus which anaesthesia is associated with less incidence of postoperative delirium for older patients undergoing hip fracture surgery. We aim to assess whether regional anaesthesia result in lower incidence of post-operative delirium comparing to general anaesthesia among older patients undergoing hip fracture surgery.

Methods and analysis:
RAGA-delirium is a pragmatic, multicentre, prospective, parallel grouped, randomized controlled clinical trial comparing regional or general anaesthesia for hip fracture surgery.
A total of 1000 patients over 65 years old who are planned to have hip fracture surgery in nine clinical trial centres of China will be randomized in a 1:1 ratio to receive either anaesthesia for the surgery.
The primary endpoint will be the incidence of postoperative delirium at day7. The secondary endpoints will be the subtype, severity and duration of delirium, postoperative acute pain score, incidence of other post-operative non-delirium complications, quality of life and cost effective outcomes.
Randomisation will be performed at the patient level using computer-generated assignment. Outcome assessors will be blinded from intervention assignment. Assessments will be conducted before surgery, intraoperatively, post-operatively, during the hospital stay, at 30-day, 6-month and at 1-year postoperative period.

Potential impact of study:
This study will provide clinical evidence by developing research with a more robust methodology to help anaesthetists in selecting appropriate anaesthesia for older patients with high risk for POD. At the rapidly aging era of China, the results may contribute to future national guideline.

Ethics and dissemination:
Ethical approved by the local Institutional Review Board (IRB). Trial results will be presented at national and international academic conferences, and published in peer-reviewed journals.

For peer review only - http://bmjopen.bmj.com/site/about/guidelines.xhtml

The trial has been registered on ClinicalTrials.gov (Number: NCT02213380).

Strengths and limitations

This will be a randomised controlled trial with a more robust methodology to improve trial accuracy and decrease risk of potential bias.

Methodology strengths of this trial include: internet central randomization, computer-generated assignment, blinded assessment and concealment allocation, appropriate sample size estimation,

The other strength is the pragmatic and multi-centre design of nine study sites in order to depict the outcomes of anaesthesia methods in real-life routine clinical practice.

The pragmatic and multi-centre design may limit the consistency of interventions, however. This compromise of sample and measurement homogeneity is expected to be diluted with large sample size.

Keywords: Hip fracture, Postoperative delirium, general anaestheia, regional anaesthesia

Word count (excluding title page, references, figures and tables): 4438 words

BACKGROUND

Delirium, sometimes called "acute confusional state", is a common clinical syndrome characterised by disturbed consciousness and a change in cognitive function or perception which develops over a short period of time (usually 1-2 days). [1] Postoperative delirium (POD) is a common postoperative complication, especially in the elderly, and is associated with increased mortality, longer length of hospital stay, extra nursing requirements and increased healthcare cost. [2] Currently there is no robust evidence demonstrating the efficacy of any specific treatment for POD, creating the urgent need for a research trial to investigate delirium prevention. The aetiology of delirium remains poorly understood, but studies have highlighted some pre-existing and precipitating factors such as anaesthetic agents that may pre-dispose to post- operative delirium. [3]

Anaesthesia may be classified into regional anaesthesia (RA) or general anaesthesia (GA). GA involves inducing sleep or loss of consciousness and is achieved by either inhalational agents or intravenous anaesthetic agents. RA involves an injection of local anaesthetic inside the spine (neuroaxial block) or around the nerves (peripheral nerve block) to prevent pain in the leg with the hip fracture. Older patients with hip fracture can be frail and often have many multiple medical comorbidities associated with advanced age[4], which places them at high risk of morbidity and mortality after anaesthesia. There is no clear consensus as to whether which anaesthesia can lead to improved patient outcomes following a surgical procedure. At present both RA and GA are administered during surgery for elderly people. However, the

For peer review only - http://bmjopen.bmj.com/site/about/guidelines.xhtml

eventual choice of anaesthetic regimen used is based on the preference and experience of the anaesthetist alongside discussion with the patient and their carers rather than evidence-based decision making. [5] Recently, a Cochrane systematic review based on six studies including a total of 624 participants claimed no difference in the risk of acute confusional state was found between different anaesthetic regimens. However, these reviews were based on low quality evidence for example, potential confounding like poor allocation concealment, assessor blinding or small sample sizes, [6]thus any estimate of effect still remain uncertain.

Over the last 20 years, the elderly population is growing fast in most parts of the world[7,8], and the number of older people undergoing surgical procedures has increased. [9,10] Hip fractures are a global health problem, with over 1.6 million patients suffering hip fractures worldwide each year [11,12], of which 680,000 occur amongst the 70 million elderly people in China.[13,14,15,16] It is projected that more than about 50% of total global hip fractures will occur in Asia by the year 2050. [17]The majority of people with hip fracture are elderly and are treated surgically, which requires anaesthesia. [18] However, no studies have yet investigated the effect of regional anaesthesia and general anaesthesia on the POD in elderly patients undergoing hip fracture surgery in China. The selection of an optimal anaesthesia regimen that can achieve the ideal anaesthetic effect during operation whilst reducing the influence on postoperative brain function in elderly patients is a challenge for anaesthetists, and calls for a high quality multicentre clinical trial.

METHODS AND DESIGN

Study design

This is a pragmatic, multicentre, prospective, parallel grouped, randomized controlled clinical trial with cost-effective analysis. We follow the SPIRIT (Standard Protocol Items for Randomized Trials) statement. [19] Figure 1 shows the design of the study. This protocol was written in accordance to the SPIRIT 2013 Statement (Defining standard protocol items for clinical trials) checklist. [20]

Objectives

Primary Objective

The aim of the RAGA trial is to determine whether different types of anaesthesia (RA vs GA) given to older patients undergoing hip fracture surgery result in equivalent incidence of post-operative delirium.

For peer review only - http://bmjopen.bmj.com/site/about/guidelines.xhtml

Secondary Objectives

The secondary objectives are:

- To compare the severity, type and duration of POD between the anesthesia groups;
- To assess whether there are differences in incidence of postoperative delirium in different groups of older patients (by age, dementia, or delirious patients) between groups;
- To describe the frequency of analgesics, blood pressure, pain in the post-operative period after either RA or GA, and explore other possible factors associated with increased risk of POD;
- To compare the occurrence of non-delirium complications between the groups;
- To assess and compare cost-effectiveness of utilizing RA or GA in terms of medical costs;
- To follow up and analyze long term outcomes and time to event data such as long term cognitive dysfunction, quality of life and other mortality & morbidity indices after POD during hospital stay and twelve months after hospital admission.

Participants

Study setting

This study will take place at nine hospitals in different regions of Mainland China. The hospitals have an annual total of 285,000 annual surgeries, of which 4580 were planned surgery for fragility hip fracture, with 2390 patients over 65 years old per year.

The nine investigational centres are: Department of Anaesthesiology, The Second Affiliated Hospital and Yuying Children Hospital of Wenzhou Medical University, Wenzhou, China; Department of Anaesthesiology, Tongji Hospital, Tongji Medical college, Huazhong University of Science & Technology, Wuhan, China; Department of Anaesthesiology, The Central Hospital of Lishui City, Lishui, China; Department of Anaesthesiology, Lishui People's Hospital, Lishui, China; Department of Anaesthesiology, The Second Hospital of Ningbo, Ningbo, China; Department of Anaesthesiology, The sixth Hospital of Ningbo, Ningbo, China; Department of Anaesthesiology, The First Affiliated Hospital of Nanchang University, Nanchang, China; Department of Anaesthesiology, Taizhou Enze Hospital, Taizhou, China; Department of Anaesthesiology, The First Affiliated Hospital of Wenzhou Medical University, Wenzhou, China.

Study population

For peer review only - http://bmjopen.bmj.com/site/about/guidelines.xhtml

Inclusion criteria:

- Patients ≥ 65 years
- Hospital admission for surgery for fragility hip fracture (Fragility hip fractures are defined as femoral neck, femoral head, Intertrochanteric or the subtrochanteric fractures.)
- ASA I-IV;
- The ability to receive written informed consent from the patient or patient's legal representative.

Exclusion criteria: Patients presenting with one of the following criteria will not be included in the trial:

- Multiple trauma, multiple fractures or other fractures outside the inclusion criteria, such as pathological fractures, pelvic fractures, femur fractures;
- Contraindication for general anaesthesia(drug allergies to general anaesthesia or any other drugs administered during this trial);
- Contraindication for regional anaesthesia (infection at the site of needle insertion, coagulopathy, international normalized ratio >1.4, platelet count <80×10^9 $litre^{-1}$, allergy to local anaesthetics);
- Susceptibility to malignant hyperthermia;
- Current enrollment in a clinical trial of Investigational medicine (CTIMP)

Outcomes and measurements:

Primary outcome

- The primary outcome is the incidence of postoperative delirium during 7 postoperative days, diagnosed with Confusion Assessment Method (CAM).[21]

Secondary Outcomes:

- The subtype, severity and duration of delirium in 7 days after the surgery, diagnosed with the Delirium Rating Scale-Revised-98 (DRS-R-98)[22] and CAM;
- Duration of delirium, measured by days from the onset of delirium symptom to resolution of symptoms;

For peer review only - http://bmjopen.bmj.com/site/about/guidelines.xhtml

- The intensity of postoperative pain, measured with acute pain score in 7 days after the surgery using visual analogue scale (VAS)[23];
- The occurrence of non-delirium in-hospital complications within 30 days after surgery, including chest infection, myocardial infarction, renal failure, gastrointestinal ileus;
- Length of hospital stay, measured by the sum of inpatient days from admission to discharge;
- Mortality during hospital stay and during follow-up period after hospital admission;
- Incidence of delirium in clinic or at residence 30 day, 6 month, 12 month after surgery, diagnosed with CAM;
- Cognitive and functional decline 30 day, 6 month, 12 month after surgery, diagnosed with mini-mental state examination (MMSE) and Hospital Anxiety and depression scale, HADS;
- Quality of life 30 day, 6 month, 12 month after surgery, using SF-36 questionnaire;
- Economic parameters will include total cost in hospital and expenditure for anaesthesia, and resource using costs, including equipment and disposables required for each anaesthetic technique.

Assignment of interventions

Randomisation

Randomisation will be performed at the patient level. Consecutively screened and eligible patients will be included in the trial at each center after initiation of the study. Patients will be allocated in a 1:1 ratio into either RA group or GA group one day before surgery using computer-generated assignment (web or telephone developed by the study data management provider). Stratification will be performed to minimize group imbalances on these variables by the following factors: age (65-79, ≥80), presence of pre-operative delirium (yes, no), per-operative dementia (yes, no). The investigator or designee will complete a randomization worksheet as detailed in the study manual.

Blinding

Owing to the nature of the study, it is not possible for the surgeon and anaesthetist delivering the interventions to be blinded to the treatment options. [24] In order to minimise any potential bias, investigators have been divided into different teams:

Un-blinded: The anaesthetist and other surgical staff who work in the operation theatre will know whether the participant have general or regional anaesthesia. However, these staff will not be involved in the assessment of outcome measures.

For peer review only - http://bmjopen.bmj.com/site/about/guidelines.xhtml

Blinded: Principal Investigators (PIs), Co-Investigators (Co-Is) and Statistician will not know patient's group allocation. Data collectors or outcome assessors, e.g. the medical staff who provide post-operative care in the ward and visit patients for preoperational assessment, hospital visits and subsequent follow-ups, will be blinded from group allocation throughout the study.

Implementation and data collection

Treatment and comparator

There will be two arms of research: Regional Anaesthesia group (RA group) and General Anaesthesia group (GA group). Conducting of interventions will follow the national or local routine clinical practice in China. For an overview of the schedule see Fig. 1.

Due to the nature of a pragmatic trial with only a few exclusion criteria, no further efforts are proposed to strictly standardize the applied interventions. In both groups, patients will receive anaesthesia according to local practice. The type of medication, dosage, and additional pain medication will be according to clinical protocols of each study centre and be documented in detail in the medical record and the Case Record Forms (CRF). Experienced and qualified anesthetists will be designated to perform the RA or GA. Prior to the study, study personnel are trained to follow the study protocol in accordance with the Good Clinical Practice (GCP) principles.

Recruitment and grouping

Patients will be recruited through the department of orthopaedics. All possible candidate patients for the trial will be provided with information sheets and informed about the aim of the trial, the treatment allocations, the workflow and the randomization procedure. Written informed consent will be obtained from patients in the first instance. If the patient is assessed and deemed not have the capacity to provide written informed consent then their legally authorized representative and/or caregivers will be approached to give agreement on behalf of the patient according to GCP.

During the screening visit (between 1 week and 1 day before operation), the inclusion and exclusion criteria will be assessed. If all criteria are fulfilled and informed consent is given, the patient will be included as candidates. Trained members of the research team will enrol participants and implement the assignment of participants to either anaesthesia groups according to the centre-randomization the day before surgery. Group assignment will be

For peer review only - http://bmjopen.bmj.com/site/about/guidelines.xhtml

revealed to members of the anaesthetic team only when the patient enters the operating room.

Pre-anaesthetic assessment

After a patient has given informed consent, pre-anaesthetic assessment will be conducted including demographics, medical history, physical examination, cognitive status, comorbidities, current medication use and assessment of preoperative quality of life. Then a specific form will be documented within 24 hours before surgery.

Data collectors will visit recruited patients from pre-anaesthetic assessment of the day before surgery to 7 days after surgery, discharge and follow up patients at 6 and 12 months after the surgery to collect required data. Data collectors will receive specific training of using of CAM, Delirium Rating Scale-Revised-98 (DRS-R-98), MMSE and other tests used in this trial and will be blinded to the group allocation.

Premedication for anaesthesia will not be encouraged before surgery. Any medication impairing cognitive function will be avoided. If there is any premedication for anaesthesia administered, it must be recorded in detail.

Anaesthesia procedure

General Anaesthesia group (GA group): For patients assigned to receive GA, anesthesia will be induced with propofol (or etomidate), sufentanil (or fentanyl) and will be maintained with either intravenous (propofol), inhalational (sevoflurane or isoflurane with or without nitrous oxide) through laryngeal mask airway, tracheal intubation, mechanical ventilation or ventilation, or combined intravenous-inhalational anaesthetics. Iliac fascia "3in1" including femoral nerve block or posterior lumbar plexus block, single nerve block or continuous nerve block, are recommended blocking methods.

Regional Anaesthesia group (RA group): Regional anaesthetic techniques for the surgery include epidural anaesthesia, spinal anaesthesia, and combined spinal and epidural, with or without additional peripheral nerve block according to anaesthetist experience. The type and dosage of local anaesthetic depend on anaesthetist experience too.

On the day of surgery, data collection will be performed and recorded with assessment of intraoperative and perioperative parameters as well as serious adverse events (SAEs). Detailed perioperative data including duration of anaesthesia, doses of sedatives, anesthetics and analgesics used during anesthesia, type and duration of surgery, intraoperative fluid balance and transfusion of blood products, use of drugs with

For peer review only - http://bmjopen.bmj.com/site/about/guidelines.xhtml

anticholinergic properties as well as post-operative analgesic consumption will be collected and recorded.

Postoperative observation and following-up

Following surgery, patients will be sent to the post-anaesthesia care unit (PACU) of each clinical centre for continuous routine vital signs monitoring, they will be discharged from PACU to the orthopaedic ward after they are accessed to have recovered from anaesthesia. Both groups will receive routine post-operative care on orthopaedic ward. Postoperative analgesia such as intravenous, epidural, nerve block analgesia, and oral analgesics can be administered, according to the local procedures of each clinical trial site, aiming to maintain a VAS pain score ≤ 30 mm, but any post-operative sedatives will be avoided.

During the postoperative visits, the primary and secondary endpoints, Adverse Events and Serious Adverse Events will be assessed and documented, including CAM, DRS-98-R (if applicable), VAS, analgesic use (if applicable), sedative use (if applicable), post-operative morbidity and laboratory results (including serum haemoglobin, haematocrit, leucocytes, aspartate aminotransferase (AST), alanine aminotransferase (ALT), albumin, serum creatinine and urea concentrations, serum sodium and potassium and serum glucose concentration.

All randomized patients will be followed up for 7 days after surgery (or until discharge from the hospital), with a daily delirium assessment. For patients who are not delirious on day seven, daily assessments are stopped. For patients who are delirious by day seven, daily clinical assessments are continued to evaluate duration of delirium until the symptoms of delirium are resolved or until the patient is discharged.

Adverse events or severe adverse event

An adverse event (AE)[25] indicates any unpredictable, unfavorable medical event that is associated with any medical intervention and occurs during the study period. It can be related to the study intervention or otherwise. A serious adverse event (SAE) in the RAGA trial is defined as any unpredictable medical events that causes death, life threatening, results in significant incapacity/disability requires prolonged hospitalisation or is otherwise considered medically significant by the investigators.

Adverse events will be reported in accordance with the guidance of good clinical practice. Information of all AE , including the type, date(s) of onset and resolution (if applicable), severity of influence, relationship with grouping, treatment and outcome will be recorded in

For peer review only - http://bmjopen.bmj.com/site/about/guidelines.xhtml

the Case Report Forms, monitored carefully, managed promptly according to routine practice, and followed up until they are properly resolved, stabilized or recovered to normal.

Concomitant treatment

The medication concomitant with the RA or GA should be as simple as possible. Previous study reports that several drugs may increase the risk of POD. [26,27,28] So the following medications such as intraoperative benzodiazepines are prohibited. Additionally, opioids (remifentanil, sufentanil, fentanyl, or morphine) and muscle relaxant (rocuronium, atracurium, or cisatracurium) will be administered when deemed clinically necessary by the attending anesthesiologists or physicians. Dosage, route, unit frequency of administration, and indication for administration and dates of all concomitant medication should be captured and recorded in details if used in clinical necessity.

Study Withdrawal

A patient will be withdrawn from the trial for any of the following reasons. The reason for the participant being withdrawn from the trial will be recorded on the 'withdrawal/change of status' form:

1. Participants choose to withdraw consent;
2. If continuing the trial is harmful to the patient's well-being;
3. The participant becomes unable to complete the trial documentation and the trial investigators feel it is no longer appropriate for the participant to continue;
4. Serious adverse events that are deemed related to the trial interventions;
5. Safety reasons determined by the trial admission or the advisory board.

Data management

The data will be managed and analyzed according to the guideline of Good Clinical practice and handled in strictest confidence. All trial data will be recorded into a electronical case report form (eCRF) by investigator-designated and appropriately trained personnel. The eCRF must be completed as soon as possible after the source document information is collected. An explanation must be given for any missing data. A copy will be retained at the trial centre, and the original eCRF will be sent via web-based submitting system to trial coordinating centre after accuracy and completeness checking by the data monitoring staff.

For peer review only - http://bmjopen.bmj.com/site/about/guidelines.xhtml

The completed CRF must be reviewed and input into a password protected trial database with double independent entries. After checking for plausibility, consistency and completeness, the database will be exported into the statistical analysis system. Appropriate backup copies of the database and related software files will be maintained. Databases are backed up by the database administrator in conjunction with any updates or changes to the database.

Data Sharing Statement

The data gathered will be anonymised for processing. Data will be held securely and used only by the researcher and supervisor. It will be held for a maximum of three years to inform the project and will then archived in an anonymised form and deposited. All data generated during the project will be made freely available via the Research Data Repository of The Second Affiliated Hospital and Yuying Children Hospital of Wenzhou Medical University. DOIs to these data will be provided and cited in any published articles using this data and any other data generated in the project. No further security, licensing or ethical issues are related to the expected data. Any data relevant to a published article will be made available alongside the article when published.

Statistics

Sample size estimation

The sample size calculation is based on incidence rate of POD. Previous published incidence of POD diagnosed using CAM ranged from 28% to 50% in the elderly patients (age 65) during hospital stay for hip fracture surgery. Observational studies in China estimated three-day postoperative delirium incidences of 11.1% to 23.3%.[29,30,31] Assuming that the seven-day GA Group in the present study will have a similar average delirium incidence as in previous three-day studies, we adopted a conservative estimation of seven-day post-surgery POD incidence undergoing general anaesthesia of 26% and a reduction of 30% POD in group RA. Then 441 patients should be needed for each group to give power (1 - β) of 0.80 and the significance level of 0.05 (two-sided). Losses to follow-up for the primary outcome are estimated to be 10.0%. Thus, a total of 1000 patients will be recruited from the nine clinical trial centres in China.

For peer review only - http://bmjopen.bmj.com/site/about/guidelines.xhtml

Statistical analysis

Statistical analyses will be performed with SAS (Statistical Analysis System, SAS Institute, Cary, NC, USA) by the statistician team of the trial. Prior to the analysis of the final study data, a detailed Statistical Analysis Plan (SAP) will be developed to describe all analyses that will be performed. Data will be primarily analysed according to the intention-to-treat principle and additional evaluation per protocol will be compared with those of the ITT analysis.

All tests will be two-sided and statistical significance will be considered at $p < 0.05$. All parameters will be calculated and presented with estimates and 95% CIs. Differences between groups will be calculated. Primary outcome (the 7-day incidence of postoperative delirium) will be analyzed using Chi-squared tests. Continuous secondary outcome measures, if normally distributed, will be tested using Students' t-test, otherwise Mann-Whitney test will be employed. Categorical outcome variables will be tested with a Chi-square test. The time-to-event data, such as delirium-free duration or discharge form hospital will be tested using a log-rank test. Safety and tolerability data will be summarized by treatment groups and will include the number of patients, the rate of occurrence, the severity and relationship to interventions.

Possible confounding factors include demography, fracture characteristics, type of surgery and peri- and postoperative complications. Stratification will act as a fundamental adjustment method for controlling the most essential confounders. Subgroup analysis according to variables relevant to the risk of POD (age, existing cognitive impairment, preoperative delirium) will help to define the risks of POD. Baseline measurements of the outcome variables, together with factors such as age, gender, BMI, co-morbidities and patient preference, will be included as covariates. Model-adjusted odds ratios between the two treatment groups will be estimated and tested with logistic regression for adjusting baseline measures to compare differences between groups in the primary and in the secondary outcome measures.

Ethical and management considerations

The trial will be conducted in compliance with the protocol and Good Clinical Practice. Monitoring will be predefined in a study-specific monitoring manual, and will be conducted by Clinical Research Unit of The Second Affiliated Hospital and Yuying Children Hospital of Wenzhou Medical University according to approved standard operating procedures. The monitor will verify the enrolment of patients, check the informed consent forms, verify the source data & entries into the CRF, and regularly supervise the progress of the trial to assist

For peer review only - http://bmjopen.bmj.com/site/about/guidelines.xhtml

the local investigators in conducting the study according to the protocol, as well as to meet regulatory and ethical requirements. In accordance with the standard operating procedures and policies of the local Institutional Review Board (IRB)/ Independent Ethics Committee (IEC), the site investigator will report SAEs to the PI and the IRB of the Second Affiliated Hospital and Yuying Children Hospital of Wenzhou Medical University.

Approvals

The study has been approved by the Institutional Review Board (IRB) of The Second Affiliated Hospital and Yuying Children Hospital of Wenzhou Medical University and by the local IRB of the participating centres. The trial is registered at clinicaltrials.gov (NCT02213380)

Trial status

The current protocol is version 1.1. The randomized trial, which commenced in September 2014, is currently in the phase of participant enrollment and follow up. To date (31th August 2016), *968 patients have been screened and* 399 patients have been randomized in this study. Recruitment of patients is about 50% slower than expected. So the recruitment period of this trial will be extended from January 2016 to September 2017.

DISCUSSION

The RAGA delirium trial is to allow us to detect whether RA given to older patients undergoing hip fracture surgery is related to a significantly lower incidence of POD. Hip fracture will present a real social and economic challenge as the population ages and the number of fractures increases. Both RA and GA are widely used in hip fracture surgery. Currently available evidence is controversial whether GA accelerates the cognitive impairment, or whether RA reduces POD. A study examining elective surgery suggests no difference in postoperative delirium when regional and general anaesthesia are compared.[32] However, the results require verification as the study was underpowered (n=29 per group).[33] A recently published result of a clinical trial shows a contradictive conclusion that the 30-day mortality was marginally reduced for spinal anaesthesia 7/164 (4.3%) versus 5/158 (3.2%) (p = 0.57), whilst at 1 year it was lower for general anaesthesia 20/163 (12.1%) versus 32/158 (20.2%) (p = 0.05). Methodological rigour in particular regarding randomization allocation concealment and assessor blinding of these studies were

For peer review only - http://bmjopen.bmj.com/site/about/guidelines.xhtml

suboptimal[5]. The numbers of participants included are insufficient to eliminate a difference between the two techniques in the majority of outcomes studied. Large randomized trials reflecting actual clinical practice are required before drawing final conclusions.

Strengths and limitations

In the present trial, a more robust methodology is adopted to decrease risk of potential bias. Firstly, we are one of the few academic clinical research teams equipped with web-based central randomization in China.

1. Utilization of computer-generated assignment to assign randomisation to recruited patients to each group; additionally covariate adaptive randomisation is also possible using the method of minimization to assess the imbalance of sample size among several covariates.
2. Clinical trials display greater difficulty instituting ‘‘blinding'', we require data collectors and outcome assessors to remain blinded to group allocation in order to prevent subsequent differential co-interventions or biased assessment of outcomes to minimise performance bias and a Hawthorne effect.[24]~~[23]~~ These measures ensure grouping balance against bias in all the respect except the intervention each group received[34].
3. Other strength is the pragmatic and multicentre design. We adopted a pragmatic design with the aim of depicting the outcomes of anaesthesia methods in real-life routine clinical practice which are more representable of clinical practice and producing results that can be generalized. Therefore, the anaesthesia methods for both groups will not be restricted to one or two specific methods in the trail.
4. On the other hand, however, the pragmatic and multicentre design may limit the accuracy of interventions measurement and the plausibility of comparison. Unlike randomized clinical trial for medications, there is variation in interventions performed by anaesthetists in different centres due to the operational characteristics of anaesthesia. Furthermore, there is variability in the surgical interventions performed in various centres due to surgeon variability and numerous other variables. This compromise of sample and result homogeneity could introduce biases influencing both arms but their effect will be diluted with large sample size.

Conclusion

In conclusion, this study will provide clinical evidence on the safety of regional and general anaesthesia in older patients for hip fracture surgery. We propose to contribute to the emerging literature by developing a more robust pragmatic multicentre randomized controlled clinical trial. The expected results of the trial are expected to help anaesthetists in

For peer review only - http://bmjopen.bmj.com/site/about/guidelines.xhtml

selecting appropriate anaesthesia in a specific subgroup of patients and especially those at high risk for POD.

Funding

This work was funded by Health and Family Planning Commission of Zhejiang Province, China(Grant No. 2014PYA015) and funded by The Recruitment Program of Global Experts, China.

Contributors:

All authors have made substantial contributions to the conception or design of the work, or the acquisition, analysis or interpretation of data. TL and YZ drafted the manuscript. JY, JL, YG, TM, NW, QQL and FG revised it critically for important intellectual content. All authors approved final version and agreed to be accountable for all aspects of the work in ensuring that questions related to the accuracy or integrity of any part of the work are appropriately investigated and resolved.

Declaration of interest

None declared.

Figure 1 Flow diagram of the RAGA trial (Please see the supplementary .tif file.)

[1] National Clinical Guideline Centre (UK). DELIRIUM: diagnosis, prevention and management [Internet]. London: Royal College of Physicians (UK) 2013. (NICE Clinical Guidelines, No. 103.). Available from: https://www.nice.org.uk/guidance/CG103

[2] S. Deiner, J. H. Silverstein. Postoperative delirium and cognitive dysfunction. Br J Anaesth. 2009; 103(Suppl 1): i41–i46. doi: 10.1093/bja/aep291

[3] Sharon K. Inouye,Thomas Robinson,Caroline Blaum,Jan Busby-Whitehead,Malaz Boustani,Ara Chalian,Stacie Deiner,Donna Fick,Lisa Hutchison,Jason Johanning,Mark Katlic,James Kempton,Maura Kennedy,Eyal

For peer review only - http://bmjopen.bmj.com/site/about/guidelines.xhtml

Kimchi,Cliff Ko,Jacqueline Leung,Melissa Mattison et al. Postoperative Delirium in Older Adults: Best Practice Statement from the American Geriatrics Society. Journal of the American College of Surgeons 2015; 220(2)136–148.e1. DOI: http://dx.doi.org/10.1016/j.jamcollsurg.2014.10.019

[4] Henderson, C.Y. & Ryan, J.P. Predicting mortality following hip fracture: an analysis of comorbidities and complications. Irish Journal of Medical Science 2015; 184(3): 667-671. doi:10.1007/s11845-015-1271-z

[5] National Clinical Guideline Centre (UK). The Management of Hip Fracture in Adults [Internet]. London: Royal College of Physicians (UK); 2011. (NICE Clinical Guidelines, No. 124.). Available from: https://www.nice.org.uk/guidance/cg124.

[6] Guay J, Parker MJ, Gajendragadkar PR, Kopp S. Anaesthesia for hip fracture surgery in adults. Cochrane Database of Systematic Reviews 2016, Issue 2. Art. No.: CD000521. DOI: 10.1002/14651858.CD000521.pub3.

[7] Dhanwal DK, Dennison EM, Harvey NC, Cooper C. Epidemiology of hip fracture: Worldwide geographic variation. Indian Journal of Orthopaedics. 2011;45(1):15-22. doi:10.4103/0019-5413.73656.

[8] Kanonidou Z, Karystianou G. Anesthesia for the elderly. Hippokratia. 2007;11(4):175-177. PMCID: PMC2552979

[9] Klopfenstein, C. E., Herrmann, F. R., Michel, J. P., Clergue, F., & Forster, A. (1998). The Influence of an Aging Surgical Population on the Anesthesia Workload. Anesthesia & Analgesia, 86(6), 1165–1170. doi:10.1097/00000539-199806000-00005

[10] Etzioni, D. A., Liu, J. H., Maggard, M. A., & Ko, C. Y. (2003). The Aging Population and Its Impact on the Surgery Workforce. Annals of Surgery, 238(2), 170–177. doi:10.1097/01.sla.0000081085.98792.3d

[11] Cummings, S. R., & Melton, L. J. (2002). Epidemiology and outcomes of osteoporotic fractures. The Lancet, 359(9319), 1761–1767. doi:10.1016/s0140-6736(02)08657-9

[12] Autier, P., Haentjens, P., Bentin, J., Baillon, J. M., Grivegnée, A. R., Closon, M. C., & Boonen, S. (2000). Costs Induced by Hip Fractures: A Prospective Controlled Study in Belgium. Osteoporosis International, 11(5), 373–380. doi:10.1007/s001980070102

[13] Wei-Bo Xia ,1 Shu-Li He,1 Ling Xu,2 Ai-Ming Liu ,3 Yan Jiang ,1 Mei Li ,1 Ou Wang,1 Xiao-Ping Xing,1

Yue Sun,1 and Steven R. Cummings. Rapidly Increasing Rates of Hip Fracture in Beijing, China. Journal of Bone and Mineral Research. 2012 Jan;27(1):125-9. DOI: 10.1002/jbmr.519

[14] Cong, E. and M.D. Walker, The Chinese skeleton: insights into microstructure that help to explain the epidemiology of fracture. 2014. 2: 14009. doi:10.1038/boneres.2014.9

[15] Wang J, Wang Y, Liu W-D, Wang F, Yin Z-S. Hip fractures in Hefei, China: the Hefei osteoporosis project. Journal of Bone and Mineral Metabolism [Internet]. Springer Nature; 2013 Jun 20;32(2):206–14. doi:10.1007/s00774-013-0484-3

[16] Chau PH, Wong M, Lee A, Ling M, Woo J. Trends in hip fracture incidence and mortality in Chinese population from Hong Kong 2001-09. Age and Ageing [Internet]. Oxford University Press (OUP); 2012 Nov 29;42(2):229–33. doi: 10.1093/ageing/afs177

[17] United Nations, Department of Economic and Social Affairs, Population Division. World Population Ageing. 2015. Available from: http://www.un.org/en/development/desa/population/publications/pdf/ageing/WPA2015_Report.pdf

[18] Guay J, Parker MJ, Gajendragadkar PR, Kopp S. Anaesthesia for hip fracture surgery in adults. Guay J, editor. Cochrane Database of Systematic Reviews [Internet]. Wiley-Blackwell; 2016 Feb 22; Available from: http://dx.doi.org/10.1002/14651858.cd000521.pub3

[19] Dutton SJ. The SPIRIT 2013 Statement. Maturitas [Internet]. Elsevier BV; 2014 May;78(1):1–2. doi:10.1016/j.maturitas.2014.02.008

[20]Chan A-W, Tetzlaff JM, Gøtzsche PC, Altman DG, Mann H, Berlin J, Dickersin K, Hróbjartsson A, Schulz KF, Parulekar WR, Krleža-Jerić K, Laupacis A, Moher D. SPIRIT 2013 Explanation and Elaboration: Guidance for protocols of clinical trials. BMJ 2013;346:e7586. doi: https://doi.org/10.1136/bmj.e7586

[21] Inouye, S. K. (1990). Clarifying Confusion: The Confusion Assessment Method. Annals of Internal Medicine, 113(12), 941. doi:10.7326/0003-4819-113-12-941

[22] Trzepacz, P. T. (2001). Validation of the Delirium Rating Scale-Revised-98: Comparison With the Delirium Rating Scale and the Cognitive Test for Delirium. Journal of Neuropsychiatry, 13(2), 229–242. doi:10.1176/appi.neuropsych.13.2.229

[23] Huskisson EC. Measurement of pain. J Rheumatol 1982;9:768-9. PMID: 6184474

[24] Karanicolas PJ, Farrokhyar F, Bhandari M. Blinding: Who, what, when, why, how? Canadian Journal of Surgery. 2010;53(5):345-348. PMCID: PMC2947122

[25] Liauw WS1, Day RO. Adverse event reporting in clinical trials: room for improvement Med J Aust. 2003;179(8):426-8. PMID: 14558867

[26] Hammon K, De Martino K: Postoperative delirium secondary to atropine premedication.AnesthProg 1985, 32(3):107-108. PMCID: PMC2148511

For peer review only - http://bmjopen.bmj.com/site/about/guidelines.xhtml

[27] Carpenter, C. R. (2011). Review: Insufficient evidence exists about which drugs are associated with delirium; benzodiazepines may increase risk. Annals of Internal Medicine, 154(12), JC6. doi:10.7326/0003-4819-154-12-201106210-02010

[28] Adunsky, A., Levy, R., Heim, M., Mizrahi, E., & Arad, M. (2002). Meperidine analgesia and delirium in aged hip fracture patients. Archives of Gerontology and Geriatrics, 35(3), 253–259. doi:10.1016/s0167-4943(02)00045-6

[29] TAN Gang, GUO Xiang-yang, LUO Ai-lun 1, HUANG Yu-guang, XU Jian-qing. Epidemiological Survey on Postoperative Delirium in Elderly Noncardiac Surgical Patients. Medical Journal of Peking Union Medical College Hospital 2011;2(4):319-325. DOI: 10. 3969 /j. issn. 1674-9081. 2011. 04. 008

[30] ZHANG Cheng-hua, MA Wei-qing,YANG Yun-li, et al. The incidence and risk factors of postoperative delirium in elderly patients undergoing hip fracture operation. J Clin Anesthesiol 2011; 27(5): 455-457. Available from: http://en.cnki.com.cn/Article_en/CJFDTOTAL-LCMZ201105017.htm

[31] WANG Bin, Z HANG Qi-liang , LI Jun, LIU Shu-yun, BI Yan-lin. Risk factors of post-operative delirium and cognitive dysfunction in elderly patients undergoing hip joint replacement surgery. J Clin Anesthesiol 2013;29(8):785-788. Available from: http://en.cnki.com.cn/Article_en/CJFDTotal-LCMZ201308028.htm

[32] Bryson GL, Wyand A. Evidence-based clinical update: General anesthesia and the risk of delirium and postoperative cognitive dysfunction. Can J Anaesth. 2006; 53(7):669–677. PubMed: 16803914

[33] Hudetz JA, Patterson KM, Iqbal Z, et al. Ketamine attenuates delirium after cardiac surgery with cardiopulmonary bypass. J Cardiothorac Vasc Anesth. 2009; 23(5):651–657. PubMed: 19231245

[34] KP Suresh. An overview of randomization techniques: An unbiased assessment of outcome in clinical research. J Hum Reprod Sci. 2011 Jan-Apr; 4(1): 8–11. doi:10.4103/0974-1208.82352

For peer review only

For peer review only - http://bmjopen.bmj.com/site/about/guidelines.xhtml

BMJ Open

Comparison of regional with general anaesthesia on post-operative delirium (RAGA-delirium) in the older patients undergoing hip fracture surgery: study protocol for a multicentre randomized controlled trial

Journal:	*BMJ Open*
Manuscript ID	bmjopen-2017-016937.R1
Article Type:	Protocol
Date Submitted by the Author:	08-Jun-2017

Complete List of Authors:	Li, Ting; The Second Affiliated Hospital and Yuying Children hospital of Wenzhou Medical University<br>Yeung, Joyce; University of Birmingham, College of Medical and Dental Sciences; Heart of England NHS Foundation Trust, Academic Department of Anaesthesia, Critical Care, Pain and Resuscitation<br>Li, Jun; The Second Affiliated Hospital and Yuying Children hospital of Wenzhou Medical University<br>Zhang, Yan; Fudan University; Shanghai Institute of Planned Parenthood Research (SIPPR)<br>Melody, Teresa; Heart of England NHS Foundation Trust, Academic Department of Anaesthesia, Critical Care, Pain and Resuscitation<br>Gao, Ye; The Second Affiliated Hospital and Yuying Children hospital of Wenzhou Medical University<br>Wang, Yi; The Second Affiliated Hospital and Yuying Children hospital of Wenzhou Medical University<br>Lian, Qian-Quan; The Second Affiliated Hospital and Yuying Children hospital of Wenzhou Medical University<br>Gao, Fang; The Second Affiliated Hospital and Yuying Children hospital of Wenzhou Medical University; University of Birmingham, Institute of Inflammation and Ageing, College of Medical and Dental Sciences

**Primary Subject Heading**:	Anaesthesia
Secondary Subject Heading:	Geriatric medicine
Keywords:	Hip fracture, Postoperative delirium, general anaestheia

SCHOLARONE™ Manuscripts

TITLE: Comparison of regional with general anaesthesia on post-operative delirium (RAGA-delirium) in the older patients undergoing hip fracture surgery: study protocol for a multicentre randomized controlled trial

AUTHORS: Ting Li, Joyce Yeung, Jun Li, Yan Zhang, Teresa Melody, Ye Gao, Yi Wang, Qianquan Lian, Fang Gao*, on behalf of the RAGA-Delirium Investigators

Ting Li:
1 The Second Affiliated Hospital and Yuying Children hospital of Wenzhou Medical University, Wenzhou, Zhejiang, CN

Joyce Yeung:
1 University of Birmingham, College of Medical and Dental Sciences, Birmingham, UK
2. Heart of England NHS Foundation Trust, Academic Department of Anaesthesia, Critical Care, Pain and Resuscitation, Birmingham Heartlands Hospital, Birmingham, UK

Jun Li:
1 The Second Affiliated Hospital and Yuying Children hospital of Wenzhou Medical University, Wenzhou, Zhejiang, CN

Yan Zhang:
1. Fudan University, Shanghai, CN
2. Shanghai Institute of Planned Parenthood Research (SIPPR),Shanghai, CN

Teresa Melody:
1. Heart of England NHS Foundation Trust, Academic Department of Anaesthesia, Critical Care, Pain and Resuscitation, Birmingham, UK

Ye Gao:
1 The Second Affiliated Hospital and Yuying Children hospital of Wenzhou Medical University, Wenzhou, Zhejiang, CN

Yi Wang:
1 The Second Affiliated Hospital and Yuying Children hospital of Wenzhou Medical University, Wenzhou, Zhejiang, CN

Qianquan Lian:
1 The Second Affiliated Hospital and Yuying Children hospital of Wenzhou Medical University, Wenzhou, Zhejiang, CN

Prof. Fang Gao (*corresponding author):
1. The Second Affiliated Hospital and Yuying Children hospital of Wenzhou Medical University, Wenzhou, Zhejiang, CN

For peer review only - http://bmjopen.bmj.com/site/about/guidelines.xhtml

2. University of Birmingham, Institute of Inflammation and Ageing, College of Medical and Dental Sciences, Birmingham, UK
Postal Address: Queen Elizabeth Hospital, University of Birmingham, Birmingham, B15 2WB, UK
Tel: +44 (0) 121 424 2966
Fax: +44 (0) 121 424 2971
Email: F.GaoSmith@bham.ac.uk

ABSTRACT

Introduction:
Postoperative delirium (POD) is a common serious postoperative complication especially in older people, and is associated with increased mortality, morbidity and healthcare costs. There is no clear consensus which anaesthesia is associated with less incidence of POD for older patients. We aim to assess whether regional anaesthesia result in lower incidence of POD comparing to general anaesthesia among older patients undergoing hip fracture surgery.

Methods and analysis:
RAGA-delirium is a pragmatic, multicentre, prospective, parallel grouped, randomized controlled clinical trial comparing regional or general anaesthesia for hip fracture surgery. A total of 1000 patients over 65 years old who are planning to have hip fracture surgery in nine clinical trial centres of China will be randomized in a 1:1 ratio to receive either anaesthesia for the surgery.
The primary endpoint will be the incidence of postoperative delirium at day7. The secondary endpoints will be the subtype, severity and duration of delirium, postoperative acute pain score, incidence of other post-operative non-delirium complications, quality of life and cost effective outcomes.
Randomisation will be performed at the patient level using computer-generated assignment. Outcome assessors will be blinded from intervention assignment. Assessments will be conducted before surgery, intraoperatively, post-operatively, during the hospital stay, at 30-day, 6-month and at 1-year postoperative intervals.

Potential impact of study:
This study will provide clinical evidence with a more robust methodology to help anaesthetists in selecting appropriate anaesthesia for older patients with high risk for POD. At the era of increasing emphasis on delirium prevention, this trial has the potential to inform the future national guideline to reduce POD.

Ethics and dissemination:
Ethical approved by the local Institutional Review Board (IRB). Trial results will be presented at national and international academic conferences, and published in peer-reviewed journals.
The trial has been registered on ClinicalTrials.gov (Number: NCT02213380).

For peer review only - http://bmjopen.bmj.com/site/about/guidelines.xhtml

Strengths and limitations

This will be a randomised controlled trial with a more robust methodology to improve trial accuracy and decrease risk of potential bias.

Methodology strengths of this trial include: internet central randomization, computer-generated assignment, blinded assessment and concealment allocation, and appropriate sample size estimation,

The other strength is the pragmatic and multi-centre design of nine study sites in order to depict the outcomes of anaesthesia methods in real-life routine clinical practice.

The pragmatic and multi-centre design may limit the consistency of interventions, however. This compromise of sample and measurement homogeneity is expected to be diluted with large sample size.

Keywords: Hip fracture, Postoperative delirium, general anaestheia, regional anaesthesia

Word count (excluding title page, references, figures and tables): 4438 words

BACKGROUND

Postoperative delirium (POD) is a common postoperative complication, especially in the elderly, and is associated with increased mortality, posttraumatic stress disorder, longer length of hospital stay, extra nursing requirements and increased healthcare cost. [1,2]Systematic reviews have shown a high incidence of delirium in surgery after hip fracture (4-53%).[3]Currently there is no robust evidence demonstrating the efficacy of any specific treatment for POD, creating the urgent need for researches to investigate delirium prevention. The aetiology of delirium remains poorly understood, but studies have highlighted some pre-existing and precipitating factors such as anaesthetic agents that may be associated with pre-dispose to post-operative delirium. [4]

Anaesthesia may be classified into regional anaesthesia (RA) or general anaesthesia (GA). GA involves inducing sleep or loss of consciousness and is achieved by either inhalational agents or intravenous anaesthetic agents. RA involves an injection of local anaesthetic inside the spine (neuroaxial block) or around the nerves (peripheral nerve block) to prevent pain in the leg with the hip fracture. Older patients with hip fracture can be frail and often have many multiple medical comorbidities associated with advanced age[5], which places them at high risk of morbidity and mortality after anaesthesia. There is no up-to-date prospective data [4]and clear consensus as to whether which anaesthesia can lead to better patient outcomes undergoing a surgery. At present both RA and GA are administered during surgery for elderly people. However, the eventual choice of anaesthetic regimen used is based on the preference and experience of the anaesthetist alongside discussion with the patient and

For peer review only - http://bmjopen.bmj.com/site/about/guidelines.xhtml

their carers rather than evidence-based decision making. [6] Recently, a Cochrane systematic review based on six studies including a total of 624 participants claimed no difference in the risk of acute confusional state was found between different anaesthetic regimens. However, current reviews were based on old and low quality evidence, such as poor allocation concealment, assessor blinding or small sample sizes, [7]thus any estimate of effect still remain uncertain.

Over the last 20 years, the elderly population is growing fast in most parts of the world[8,9], and the number of older people undergoing surgical procedures has increased. [10,11] Hip fractures are a global-health problem, with over 1.6 million patients suffering hip fractures worldwide each year [12,13], of which 680,000 occur amongst the 70 million elderly people in China.[14,15,16,17] It is projected that more than 50% of total global hip fractures will occur in Asia by the year 2050. [18]The majority of people with hip fracture are elderly and are treated surgically, which requires anaesthesia. [19] However, no studies have yet investigated the effect of regional anaesthesia and general anaesthesia on the POD in elderly patients undergoing hip fracture surgery in China. The selection of an optimal anaesthesia regimen that can achieve the ideal anaesthetic effect during operation whilst reducing the influence on postoperative brain function in elderly patients is a challenge for anaesthetists, and calls for a high quality multicentre clinical trial.

METHODS AND DESIGN

Study design

This is a pragmatic, multicentre, prospective, parallel grouped, randomized controlled clinical trial with cost-effective analysis. This follow the SPIRIT 2013 (Standard Protocol Items for Randomized Trials) statement. [20][21]Figure 1 shows the design of the study.

Objectives

Primary Objective

The aim of the RAGA trial is to determine whether different types of anaesthesia (RA vs GA) given to older patients undergoing hip fracture surgery result in equivalent incidence of post-operative delirium.

Secondary Objectives

- To compare the severity, type and duration of POD between the anesthesia groups;
- To assess whether there are differences in incidence of postoperative delirium in different patients (by age, dementia, or delirious patients) between groups;

For peer review only

- To describe the frequency of analgesics, blood pressure, pain in the post-operative period after either RA or GA, and explore other possible factors associated with increased risk of POD;
- To compare the occurrence of non-delirium complications between the groups;
- To assess and compare cost-effectiveness of utilizing RA or GA in terms of medical costs;
- To follow up and analyze long term outcomes and time to event data such as long term cognitive dysfunction, quality of life and other mortality & morbidity indices after POD during hospital stay and twelve months after hospital admission.

Participants

Study setting

This study will take place at nine hospitals in different regions of Mainland China. The hospitals have an annual total of 285,000 annual surgeries, of which 4580 were planned surgery for fragility hip fracture, with 2390 patients over 65 years old per year.

The nine investigational centres are: Department of Anaesthesiology, The Second Affiliated Hospital and Yuying Children Hospital of Wenzhou Medical University, Wenzhou, China; Department of Anaesthesiology, Tongji Hospital, Tongji Medical college, Huazhong University of Science & Technology, Wuhan, China; Department of Anaesthesiology, The Central Hospital of Lishui City, Lishui, China; Department of Anaesthesiology, Lishui People's Hospital, Lishui, China; Department of Anaesthesiology, The Second Hospital of Ningbo, Ningbo, China; Department of Anaesthesiology, The sixth Hospital of Ningbo, Ningbo, China; Department of Anaesthesiology, The First Affiliated Hospital of Nanchang University, Nanchang, China; Department of Anaesthesiology, Taizhou Enze Hospital, Taizhou, China; Department of Anaesthesiology, The First Affiliated Hospital of Wenzhou Medical University, Wenzhou, China.

Study population

Inclusion criteria:

- Patients ≥ 65 years old;
- Hospital admission for surgery for fragility hip fracture (Fragility hip fractures are defined as femoral neck, femoral head, Intertrochanteric or the subtrochanteric fractures);

For peer review only

- ASA I-IV;
- The ability to receive written informed consent from the patient or patient's legal representative.

Exclusion criteria: Patients presenting with one of the following criteria will not be included in the trial:

- Multiple trauma, multiple fractures or other fractures outside the inclusion criteria, such as pathological fractures, pelvic fractures, femur fractures;
- Contraindication for general anaesthesia(drug allergies to general anaesthesia or any other drugs administered during this trial);
- Contraindication for regional anaesthesia (infection at the site of needle insertion, coagulopathy, international normalized ratio >1.4, platelet count $<80\times10^{9}$ $litre^{-1}$, allergy to local anaesthetics);
- Susceptibility to malignant hyperthermia;
- Current enrollment in a clinical trial of Investigational medicine (CTIMP)

Outcomes and measurements:

Primary outcome

- The primary outcome is the incidence of postoperative delirium during 7 postoperative days, diagnosed with Confusion Assessment Method (CAM).[22]

Secondary Outcomes:

- The subtype, severity and duration of delirium in 7 days after the surgery, diagnosed with the Delirium Rating Scale-Revised-98 (DRS-R-98)[23] and CAM;
- Duration of delirium, measured by days from the onset of delirium symptom to resolution of symptoms;
- The intensity of postoperative pain, measured with acute pain score in 7 days after the surgery using visual analogue scale (VAS)[24];
- The occurrence of non-delirium in-hospital complications within 30 days after surgery, including chest infection, myocardial infarction, renal failure, gastrointestinal ileus;
- Length of hospital stay, measured by the sum of inpatient days from admission to discharge;
- Mortality during hospital stay and during follow-up period after hospital admission;

For peer review only - http://bmjopen.bmj.com/site/about/guidelines.xhtml

- Incidence of delirium in clinic or at residence on 30 day, 6 month, 12 month after surgery, diagnosed with CAM;
- Cognitive and functional decline on 30 day, 6 month, 12 month after surgery, diagnosed with mini-mental state examination (MMSE) and Hospital Anxiety and depression scale, HADS;
- Quality of life on 30 day, 6 month, 12 month after surgery, using SF-36 questionnaire;
- Economic parameters including total cost in hospital and expenditure for anaesthesia, and resource using costs, including equipment and disposables required for each anaesthetic technique.

Assignment of interventions

Randomisation

Randomisation will be performed at the patient level. Consecutively screened and eligible patients will be included in the trial at each center after initiation of the study. Patients will be allocated in a 1:1 ratio into either RA group or GA group one day before surgery using computer-generated assignment (web or telephone developed by the study data management provider). Stratification will be performed to minimize group imbalances on these variables by the following factors: age (65-79, ≥80), presence of pre-operative delirium (yes, no), per-operative dementia (yes, no). The investigator or designee will complete a randomisation worksheet as detailed in the study manual.

Blinding

Owing to the nature of the study, it is not possible for the surgeon and anaesthetist delivering the interventions to be blinded to the treatment options. [25] In order to minimise any potential bias, investigators have been divided into different teams:

Un-blinded: The anaesthetist and other surgical staff who work in the operation theatre will know whether the participant have general or regional anaesthesia. These staff will not be involved in the assessment of outcome measures.

Blinded: Principal Investigators (PIs), Co-Investigators (Co-Is) and Statistician will not know patient's group allocation. Data collectors or outcome assessors, e.g. the medical staff who provide post-operative care in the ward and visit patients for preoperational assessment, hospital visits and subsequent follow-ups, will be blinded from group allocation throughout the study.

For peer review only - http://bmjopen.bmj.com/site/about/guidelines.xhtml

Implementation and data collection

Treatment and comparator

There will be two arms of research: Regional Anaesthesia group (RA group) and General Anaesthesia group (GA group). Conducting of interventions will follow the national or local routine clinical practice in China. For an overview of the schedule see Fig. 1.

Due to the nature of a pragmatic trial with only a few exclusion criteria, no further efforts are proposed to strictly standardize the applied interventions. In both groups, patients will receive anaesthesia according to local practice. The type of medication, dosage, and additional pain medication will be based on clinical protocols of each study centre and be documented in detail in the medical record and in the Case Record Forms (CRF). Experienced and qualified anesthetists will be designated to perform the RA or GA. Prior to the study, study personnel are trained to follow the study protocol in accordance with the Good Clinical Practice (GCP) principles.

Recruitment and grouping

Patients will be recruited through the department of orthopaedics. All possible candidate patients for the trial will be provided with information sheets and informed about the aim of the trial, the treatment allocations, the workflow and the randomization procedure. Written informed consent will be obtained from patients in the first instance. If the patient is assessed and deemed not have the capacity to provide written informed consent then their legally authorized representative and/or caregivers will be approached to give agreement on behalf of the patient according to GCP.

During the screening visit (between 1 week and 1 day before operation), the inclusion and exclusion criteria will be assessed. If all criteria are fulfilled and informed consent is given, the patient will be included as candidates. Trained members of the research team will enrol participants and implement the assignment of participants to either anaesthesia groups according to the centre-randomization the day before surgery. Group assignment will be revealed to members of the anaesthetic team only when the patient enters the operating room.

Pre-anaesthetic assessment

After a patient has given informed consent, pre-anaesthetic assessment will be conducted including demographics, medical history, physical examination, cognitive status,

For peer review only - http://bmjopen.bmj.com/site/about/guidelines.xhtml

comorbidities, current medication use and assessment of preoperative quality of life. Then a specific form will be documented within 24 hours before surgery.

Data collectors will visit recruited patients from pre-anaesthetic assessment the day before surgery to 7 days after surgery, discharge and follow up patients at 6 and 12 months after the surgery to collect required data. Data collectors will receive specific training of using of CAM, DRS-R-98, MMSE and other tests used in this trial and will be blinded to the group allocation.

Premedication for anaesthesia will not be encouraged before surgery. Any medication impairing cognitive function will be avoided.

Anaesthesia procedure

General Anaesthesia group (GA group): For patients assigned to receive GA, anesthesia will be induced with propofol (or etomidate), sufentanil (or fentanyl) and will be maintained with either intravenous (propofol), inhalational (sevoflurane or isoflurane with or without nitrous oxide) through laryngeal mask airway, tracheal intubation, mechanical ventilation, or combined intravenous-inhalational anaesthetics. Iliac fascia "3in1", femoral nerve block or posterior lumbar plexus block, single nerve block or continuous nerve block, are recommended blocking methods.

Regional Anaesthesia group (RA group): Regional anaesthetic techniques for the surgery include epidural anaesthesia, spinal anaesthesia, and combined spinal and epidural, with or without additional peripheral nerve block according to anaesthetist experience. The type and dosage of local anaesthetic depend on anaesthetist experience as well. No sedative is administrated in RA group. Any medication impairing cognitive function, like midazolam or dexmedetomidine， will be avoided in both groups.

On the day of surgery, data collection will be performed and recorded with assessment of intraoperative and perioperative parameters as well as serious adverse events (SAEs). Detailed perioperative data including duration of anaesthesia, doses of sedatives, anesthetics and analgesics used during anesthesia, type and duration of surgery, intraoperative fluid balance and transfusion of blood products, use of drugs with anticholinergic properties as well as post-operative analgesic consumption will be collected and recorded.

For peer review only - http://bmjopen.bmj.com/site/about/guidelines.xhtml

Postoperative observation and following-up

Following surgery, patients will be sent to the post-anaesthesia care unit (PACU) of each clinical centre for continuous routine vital signs monitoring, they will be discharged from PACU to the orthopaedic ward after they are accessed to have recovered from anaesthesia. Both groups will receive routine post-operative care on orthopaedic ward. Postoperative analgesia such as intravenous, epidural, nerve block analgesia, and oral analgesics can be administered, according to the local procedures of each clinical trial site, aiming to maintain a VAS pain score ≤ 30 mm, but any post-operative sedatives will be avoided.

During the postoperative visits, the primary and secondary endpoints, Adverse Events and Serious Adverse Events will be assessed and documented, including CAM, DRS-98-R (if applicable), VAS, analgesic use (if applicable), sedative use (if applicable), post-operative morbidity and laboratory results, such as serum haemoglobin, haematocrit, leucocytes, aspartate aminotransferase (AST), alanine aminotransferase (ALT), albumin, serum creatinine and urea concentrations, serum sodium and potassium and serum glucose concentration.

All randomized patients will be followed up for 7 days after surgery (or until discharge from the hospital), with a daily delirium assessment. For patients who are not delirious on day seven, daily assessments are stopped. For patients who are delirious by day seven, daily clinical assessments are continued to evaluate duration of delirium until the symptoms of delirium are resolved or until the patient is discharged.

Adverse events or severe adverse event

An adverse event (AE)[26] indicates any unpredictable, unfavorable medical event that is associated with any medical intervention and occurs during the study period. It can be related to the study intervention or otherwise. A serious adverse event (SAE) in the RAGA trial is defined as any unpredictable medical events that causes death, life threatening, results in significant incapacity/disability requires prolonged hospitalisation or is otherwise considered medically significant by the investigators.

Adverse events will be reported in accordance with the guidance of good clinical practice. Information of all AE , including the type, date(s) of onset and resolution (if applicable), severity of influence, relationship with grouping, treatment and outcome will be recorded in the Case Report Forms, monitored carefully, managed promptly according to routine practice, and followed up until they are properly resolved, stabilized or recovered to normal.

For peer review only - http://bmjopen.bmj.com/site/about/guidelines.xhtml

Concomitant treatment

The medication concomitant with the RA or GA should be as simple as possible. Previous study reports that several drugs may increase the risk of POD. [27,28,29] So the following medications such as intraoperative benzodiazepines are prohibited. Additionally, opioids (remifentanil, sufentanil, fentanyl, or morphine) and muscle relaxant (rocuronium, atracurium, or cisatracurium) will be administered when deemed clinically necessary by the attending anesthesiologists or physicians. Dosage, route, unit frequency of administration, and indication for administration and dates of all concomitant medication should be captured and recorded in details if used in clinical necessity.

Study Withdrawal

A patient will be withdrawn from the trial for any of the following reasons. The reason for the participant being withdrawn from the trial will be recorded on the 'withdrawal/change of status' form:

1. Participants choose to withdraw consent;
2. If continuing the trial is harmful to the patient's well-being;
3. The participant becomes unable to complete the trial documentation and the trial investigators feel it is no longer appropriate for the participant to continue;
4. Serious adverse events that are deemed related to the trial interventions;
5. Safety reasons determined by the trial admission or the advisory board.

Data management

The data will be managed and analyzed according to the guideline of Good Clinical practice and handled in strictest confidence. All trial data will be recorded into a electronical case report form (eCRF) by investigator-designated and appropriately trained personnel. The eCRF must be completed as soon as possible after the source document information is collected. An explanation must be given for any missing data. A copy will be retained at the trial centre, and the original eCRF will be sent via web-based submitting system to trial coordinating centre after accuracy and completeness checking by the data monitoring staff.

The completed CRF must be reviewed and input into a password protected trial database with double independent entries. After checking for plausibility, consistency and completeness, the database will be exported into the statistical analysis system. Appropriate

For peer review only - http://bmjopen.bmj.com/site/about/guidelines.xhtml

backup copies of the database and related software files will be maintained. Databases are backed up by the database administrator in conjunction with any updates or changes to the database.

Data Sharing Statement

The data gathered will be anonymised for processing. Data will be held securely and used only by the researcher and supervisor. It will be held for a maximum of three years to inform the project and will then archived in an anonymised form and deposited.

All data generated during the project will be made freely available via the Research Data Repository of The Second Affiliated Hospital and Yuying Children Hospital of Wenzhou Medical University. DOIs to these data will be provided and cited in any published articles using the data and any other data generated in the project. No further security, licensing or ethical issues are related to the expected data. Any data relevant to a published article will be made available alongside the article when published.

Statistics

Sample size estimation

The sample size calculation is based on incidence rate of POD. Previous published incidence of POD diagnosed using CAM ranged from 28% to 50% in the elderly patients (age 65) during hospital stay for hip fracture surgery. Observational studies in China estimated three-day postoperative delirium incidences of 11.1% to 23.3%.[30,31,32] Assuming that the seven-day GA Group in the present study will have a similar average delirium incidence as in previous three-day studies, we adopted a conservative estimation of seven-day post-surgery POD incidence undergoing general anaesthesia of 26% and a reduction of 30% POD in group RA. Then 441 patients should be needed for each group to give power (1 - β) of 0.80 and the significance level of 0.05 (two-sided). Losses to follow-up for the primary outcome are estimated to be 10.0%. Thus, a total of 1000 patients will be recruited from the nine clinical trial centres in China.

Statistical analysis

Statistical analyses will be performed with SAS (Statistical Analysis System, SAS Institute, Cary, NC, USA) by the statistician team of the trial. Prior to the analysis of the final study data, a detailed Statistical Analysis Plan (SAP) will be developed to describe all analyses that

For peer review only - http://bmjopen.bmj.com/site/about/guidelines.xhtml

will be performed. Data will be primarily analysed according to the intention-to-treat (ITT) principle and additional evaluation per protocol (PP) will be compared with those of the ITT analysis.

All tests will be two-sided and statistical significance will be considered at $p < 0.05$. All parameters will be calculated and presented with estimates and 95% CIs. Differences between groups will be calculated. Primary outcome (the 7-day incidence of postoperative delirium) will be analyzed using Chi-squared tests. Continuous secondary outcome measures, if normally distributed, will be tested using Students' t-test, otherwise Mann-Whitney test will be employed. Categorical outcome variables will be tested with a Chi-square test. The time-to-event data, such as delirium-free duration or discharge form hospital will be tested using a log-rank test. Safety and tolerability data will be summarized by treatment groups and will include the number of patients, the rate of occurrence, the severity and relationship to interventions.

Possible confounding factors include demography, fracture characteristics, type of surgery and peri- and postoperative complications. Stratification will act as a fundamental adjustment method for controlling the most essential confounders. Subgroup analysis according to variables relevant to the risk of POD (age, existing cognitive impairment, preoperative delirium) will help to define the risks of POD. Baseline measurements of the outcome variables, together with factors such as age, gender, BMI, co-morbidities and patient preference, will be included as covariates. Model-adjusted odds ratios between the two treatment groups will be estimated and tested with logistic regression for adjusting baseline measures to compare differences between groups in the primary and in the secondary outcome measures.

Ethical and management considerations

The trial will be conducted in compliance with the protocol and Good Clinical Practice. Monitoring will be predefined in a study-specific monitoring manual, and will be conducted by Clinical Research Unit of The Second Affiliated Hospital and Yuying Children Hospital of Wenzhou Medical University according to approved standard operating procedures. The monitor will verify the enrolment of patients, check the informed consent forms, verify the source data & entries into the CRF, and regularly supervise the progress of the trial to assist the local investigators in conducting the study according to the protocol, as well as to meet regulatory and ethical requirements. In accordance with the standard operating procedures and policies of the local Institutional Review Board (IRB)/ Independent Ethics Committee

For peer review only - http://bmjopen.bmj.com/site/about/guidelines.xhtml

(IEC), the site investigator will report SAEs to the PI and the IRB of the Second Affiliated Hospital and Yuying Children Hospital of Wenzhou Medical University.

Approvals

The study has been approved by the Institutional Review Board (IRB) of The Second Affiliated Hospital and Yuying Children Hospital of Wenzhou Medical University and by the local IRB of the participating centres. The trial is registered at clinicaltrials.gov (NCT02213380)

Trial status

The current protocol is version 1.1. The randomized trial, which commenced in September 2014, is currently in the phase of participant enrollment and follow up. To date (31th August 2016), *968 patients have been screened and* 399 patients have been randomized in this study. Recruitment of patients is about 50% slower than expected, so the recruitment period of this trial will be extended from January 2016 to July 2018.

DISCUSSION

The RAGA delirium trial is to allow us to detect whether RA given to older patients undergoing hip fracture surgery is related to a significantly lower incidence of POD. Hip fracture will present a real social and economic challenge as the population ages and the number of fractures increases. Both RA and GA are widely used in hip fracture surgery. Currently available evidence is controversial as to whether GA accelerates the cognitive impairment, or whether RA reduces POD. A study examining elective surgery suggests no difference in postoperative delirium when regional and general anaesthesia are compared.[33] However, the results require verification as the study was underpowered (n=29 per group).[34] A recently published result of a clinical trial shows a contradictive conclusion that the 30-day mortality was marginally reduced for spinal anaesthesia 7/164 (4.3%) versus 5/158 (3.2%) (p = 0.57), whilst at 1 year it was lower for general anaesthesia 20/163 (12.1%) versus 32/158 (20.2%) (p = 0.05). Methodological rigour in particular regarding randomization allocation concealment and assessor blinding of these studies was suboptimal[6]. The numbers of participants included are insufficient to eliminate a difference between the two techniques in the majority of outcomes studied. Large randomized trials reflecting actual clinical practice are required before drawing final conclusions.

For peer review only - http://bmjopen.bmj.com/site/about/guidelines.xhtml

Strengths and limitations

In the present trial, a more robust methodology is adopted to decrease risk of potential bias. Firstly, we are one of the few academic clinical research teams equipped with web-based central randomization in China.

1. Utilization of computer-generated assignment to assign randomisation to recruited patients to each group; additionally covariate adaptive randomisation is also possible using the method of minimization to assess the imbalance of sample size among several covariates.
2. Clinical trials display greater difficulty instituting ‘‘blinding'', we require data collectors and outcome assessors to remain blinded to group allocation in order to prevent subsequent differential co-interventions or biased assessment of outcomes to minimise performance bias and a Hawthorne effect.[25] These measures ensure grouping balance against bias in all the respects except the intervention each group received[35].
3. Other strength is the pragmatic and multicentre design. We adopted a pragmatic design with the aim of depicting the outcomes of anaesthesia methods in real-life routine clinical practice which are more representable of clinical practice and producing results that can be generalized. Therefore, the anaesthesia methods for both groups will not be restricted to one or two specific methods in the trial.
4. On the other hand, however, the pragmatic and multicentre design may limit the accuracy of measurement of interventions and the plausibility of comparison. Unlike randomized clinical trial for medications, there is variation in interventions performed by anaesthetists in different centres due to the operational characteristics of anaesthesia. Furthermore, there is variability in the surgical interventions performed in various centres due to surgeon variability and numerous other variables. This compromise of sample and result homogeneity could introduce biases influencing both arms but their effect will be diluted with large sample size.

Conclusion

In conclusion, this study will provide clinical evidence on the safety of regional and general anaesthesia in older patients for hip fracture surgery. We propose to contribute to the emerging literature by developing a more robust pragmatic multicentre randomized controlled clinical trial. The results of the trial are expected to help anaesthetists in selecting appropriate anaesthesia in a specific subgroup of patients and especially those at high risk for POD.

For peer review only - http://bmjopen.bmj.com/site/about/guidelines.xhtml

For peer review only

Funding

This work was funded by Health and Family Planning Commission of Zhejiang Province, China(Grant No. 2014PYA015) and funded by The Recruitment Program of Global Experts, China.

Contributors:

All authors have made substantial contributions to the conception or design of the work, or the acquisition, analysis or interpretation of data. TL and YZ drafted the manuscript. JY, JL, YG, TM, NW, QQL and FG revised it critically for important intellectual content. TM edited the language. All authors approved final version and agreed to be accountable for all aspects of the work in ensuring that questions related to the accuracy or integrity of any part of the work are appropriately investigated and resolved.

Declaration of interest

None declared.

Figure 1 Flow diagram of the RAGA trial (Please see the supplementary .tif file.)

For peer review only

[1] S. Deiner, J. H. Silverstein. Postoperative delirium and cognitive dysfunction. Br J Anaesth. 2009; 103(Suppl 1): i41–i46. doi: 10.1093/bja/aep291

[2] Coburn, Mark; Sanders, Robert; Neuman, Mark; Rossaint, Rolf; Matot, Idit. We may have improved but we must get better still: The never-ending story of the elderly with fractured neck of femur. European Journal of Anaesthesiology,34(3): 115–117. doi: 10.1097/EJA.0000000000000560.

[3]Sanders Robert D, Pandharipande Pratik P, Davidson Andrew J, Ma Daqing, Maze Mervyn. Anticipating and managing postoperative delirium and cognitive decline in adults BMJ 2011; 343 :d4331

[4] Sharon K. Inouye,Thomas Robinson,Caroline Blaum,Jan Busby-Whitehead,Malaz Boustani,Ara Chalian,Stacie Deiner,Donna Fick,Lisa Hutchison,Jason Johanning,Mark Katlic,James Kempton,Maura Kennedy,Eyal Kimchi,Cliff Ko,Jacqueline Leung,Melissa Mattison et al. Postoperative Delirium in Older Adults: Best Practice Statement from the American Geriatrics Society. Journal of the American College of Surgeons 2015; 220(2)136–148.e1. DOI: http://dx.doi.org/10.1016/j.jamcollsurg.2014.10.019

[5] Henderson, C.Y. & Ryan, J.P. Predicting mortality following hip fracture: an analysis of comorbidities and complications. Irish Journal of Medical Science 2015; 184(3): 667-671. doi:10.1007/s11845-015-1271-z

For peer review only - http://bmjopen.bmj.com/site/about/guidelines.xhtml

[6] National Clinical Guideline Centre (UK). The Management of Hip Fracture in Adults [Internet]. London: Royal College of Physicians (UK); 2011. (NICE Clinical Guidelines, No. 124.). Available from: https://www.nice.org.uk/guidance/cg124.

[7] Guay J, Parker MJ, Gajendragadkar PR, Kopp S. Anaesthesia for hip fracture surgery in adults. Cochrane Database of Systematic Reviews 2016, Issue 2. Art. No.: CD000521. DOI: 10.1002/14651858.CD000521.pub3.

[8] Dhanwal DK, Dennison EM, Harvey NC, Cooper C. Epidemiology of hip fracture: Worldwide geographic variation. Indian Journal of Orthopaedics. 2011;45(1):15-22. doi:10.4103/0019-5413.73656.

[9] Kanonidou Z, Karystianou G. Anesthesia for the elderly. Hippokratia. 2007;11(4):175-177. PMCID: PMC2552979

[10] Klopfenstein, C. E., Herrmann, F. R., Michel, J. P., Clergue, F., & Forster, A. (1998). The Influence of an Aging Surgical Population on the Anesthesia Workload. Anesthesia & Analgesia, 86(6), 1165–1170. doi:10.1097/00000539-199806000-00005

[11] Etzioni, D. A., Liu, J. H., Maggard, M. A., & Ko, C. Y. (2003). The Aging Population and Its Impact on the Surgery Workforce. Annals of Surgery, 238(2), 170–177. doi:10.1097/01.sla.0000081085.98792.3d

[12] Cummings, S. R., & Melton, L. J. (2002). Epidemiology and outcomes of osteoporotic fractures. The Lancet, 359(9319), 1761–1767. doi:10.1016/s0140-6736(02)08657-9

[13] Autier, P., Haentjens, P., Bentin, J., Baillon, J. M., Grivegnée, A. R., Closon, M. C., & Boonen, S. (2000). Costs Induced by Hip Fractures: A Prospective Controlled Study in Belgium. Osteoporosis International, 11(5), 373–380. doi:10.1007/s001980070102

[14] Wei-Bo Xia ,1 Shu-Li He,1 Ling Xu,2 Ai-Ming Liu ,3 Yan Jiang ,1 Mei Li ,1 Ou Wang,1 Xiao-Ping Xing,1

Yue Sun,1 and Steven R. Cummings. Rapidly Increasing Rates of Hip Fracture in Beijing, China. Journal of Bone and Mineral Research. 2012 Jan;27(1):125-9. DOI: 10.1002/jbmr.519

[15] Cong, E. and M.D. Walker, The Chinese skeleton: insights into microstructure that help to explain the epidemiology of fracture. 2014. 2: 14009. doi:10.1038/boneres.2014.9

[16] Wang J, Wang Y, Liu W-D, Wang F, Yin Z-S. Hip fractures in Hefei, China: the Hefei osteoporosis project. Journal of Bone and Mineral Metabolism [Internet]. Springer Nature; 2013 Jun 20;32(2):206–14. doi:10.1007/s00774-013-0484-3

[17] Chau PH, Wong M, Lee A, Ling M, Woo J. Trends in hip fracture incidence and mortality in Chinese population from Hong Kong 2001-09. Age and Ageing [Internet]. Oxford University Press (OUP); 2012 Nov 29;42(2):229–33. doi: 10.1093/ageing/afs177

[18] United Nations, Department of Economic and Social Affairs, Population Division. World Population Ageing. 2015. Available from: http://www.un.org/en/development/desa/population/publications/pdf/ageing/WPA2015_Report.pdf

[19] Guay J, Parker MJ, Gajendragadkar PR, Kopp S. Anaesthesia for hip fracture surgery in adults. Guay J, editor. Cochrane Database of Systematic Reviews [Internet]. Wiley-Blackwell; 2016 Feb 22; Available from: http://dx.doi.org/10.1002/14651858.cd000521.pub3

[20] Dutton SJ. The SPIRIT 2013 Statement. Maturitas [Internet]. Elsevier BV; 2014 May;78(1):1–2. doi:10.1016/j.maturitas.2014.02.008

[21]Chan A-W, Tetzlaff JM, Gøtzsche PC, Altman DG, Mann H, Berlin J, Dickersin K, Hróbjartsson A, Schulz KF, Parulekar WR, Krleža-Jerić K, Laupacis A, Moher D. SPIRIT 2013 Explanation and Elaboration: Guidance for protocols of clinical trials. BMJ 2013;346:e7586. doi: https://doi.org/10.1136/bmj.e7586

[22] Inouye, S. K. (1990). Clarifying Confusion: The Confusion Assessment Method. Annals of Internal Medicine, 113(12), 941. doi:10.7326/0003-4819-113-12-941

[23] Trzepacz, P. T. (2001). Validation of the Delirium Rating Scale-Revised-98: Comparison With the Delirium Rating Scale and the Cognitive Test for Delirium. Journal of Neuropsychiatry, 13(2), 229–242. doi:10.1176/appi.neuropsych.13.2.229

[24] Huskisson EC. Measurement of pain. J Rheumatol 1982;9:768-9. PMID: 6184474

[25] Karanicolas PJ, Farrokhyar F, Bhandari M. Blinding: Who, what, when, why, how? Canadian Journal of Surgery. 2010;53(5):345-348. PMCID: PMC2947122

[26] Liauw WS1, Day RO. Adverse event reporting in clinical trials: room for improvement Med J Aust. 2003;179(8):426-8. PMID: 14558867

[27] Hammon K, De Martino K: Postoperative delirium secondary to atropine premedication.AnesthProg 1985, 32(3):107-108. PMCID: PMC2148511

[28] Carpenter, C. R. (2011). Review: Insufficient evidence exists about which drugs are associated with delirium; benzodiazepines may increase risk. Annals of Internal Medicine, 154(12), JC6. doi:10.7326/0003-4819-154-12-201106210-02010

[29] Adunsky, A., Levy, R., Heim, M., Mizrahi, E., & Arad, M. (2002). Meperidine analgesia and delirium in aged hip fracture patients. Archives of Gerontology and Geriatrics, 35(3), 253–259. doi:10.1016/s0167-4943(02)00045-6

[30] TAN Gang, GUO Xiang-yang, LUO Ai-lun 1, HUANG Yu-guang, XU Jian-qing. Epidemiological Survey on Postoperative Delirium in Elderly Noncardiac Surgical Patients. Medical Journal of Peking Union Medical College Hospital 2011;2(4):319-325. DOI: 10. 3969 /j. issn. 1674-9081. 2011. 04. 008

[31] ZHANG Cheng-hua, MA Wei-qing,YANG Yun-li, et al. The incidence and risk factors of postoperative delirium in elderly patients undergoing hip fracture operation. J Clin Anesthesiol 2011; 27(5): 455-457. Available from: http://en.cnki.com.cn/Article_en/CJFDTOTAL-LCMZ201105017.htm

[32] WANG Bin, Z HANG Qi-liang , LI Jun, LIU Shu-yun, BI Yan-lin. Risk factors of post-operative delirium and cognitive dysfunction in elderly patients undergoing hip joint replacement surgery. J Clin Anesthesiol 2013;29(8):785-788. Available from: http://en.cnki.com.cn/Article_en/CJFDTotal-LCMZ201308028.htm

[33] Bryson GL, Wyand A. Evidence-based clinical update: General anesthesia and the risk of delirium and postoperative cognitive dysfunction. Can J Anaesth. 2006; 53(7):669–677. PubMed: 16803914

[34] Hudetz JA, Patterson KM, Iqbal Z, et al. Ketamine attenuates delirium after cardiac surgery with cardiopulmonary bypass. J Cardiothorac Vasc Anesth. 2009; 23(5):651–657. PubMed: 19231245

[35] KP Suresh. An overview of randomization techniques: An unbiased assessment of outcome in clinical research. J Hum Reprod Sci. 2011 Jan-Apr; 4(1): 8–11. doi:10.4103/0974-1208.82352

For peer review only - http://bmjopen.bmj.com/site/about/guidelines.xhtml

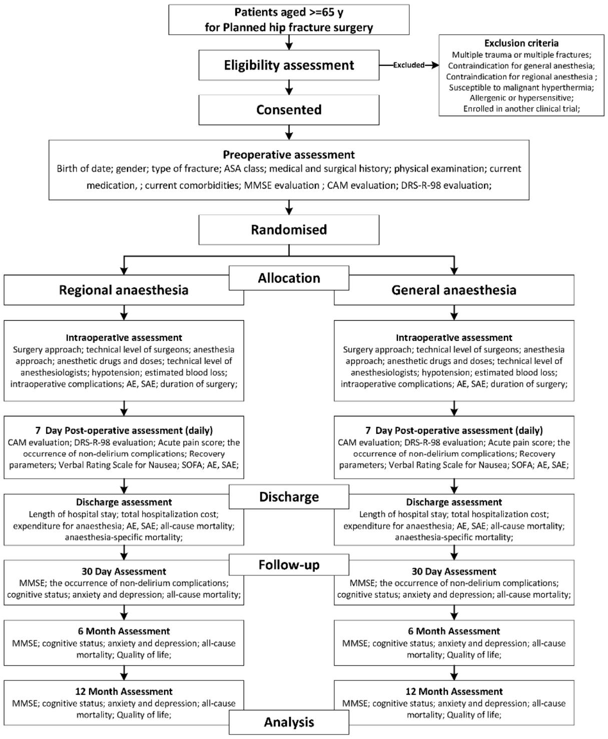

297x400mm (300 x 300 DPI)
